# Preferences of patients with multiple chronic diseases for medication in rural areas of an Eastern Province China: a discrete choice experiment

**DOI:** 10.3389/fmed.2024.1439136

**Published:** 2024-10-09

**Authors:** Xiaona Li, Dongping Ma, Zhiqiang Feng, Min Gao, Ping Dong, Yongli Shi, Ziyuan Li, Runmin Li, Wenqiang Yin, Zhongming Chen

**Affiliations:** ^1^School of Management, Shandong Second Medical University, Weifang, China; ^2^China National Health Development Research Center, Beijing, China

**Keywords:** multiple chronic diseases, patient preferences, rural areas, discrete choice experiments, medication selection, health belief model

## Abstract

**Background:**

Multiple Chronic Diseases (MCD) are the co-occurrence of two or more chronic conditions within an individual. Compared to patients with a single chronic disease, those with MCD face challenges related to polypharmacy, which increases the risk of adverse drug events, side effects, and drug–drug interactions. Understanding the specific medication preferences of patients with MCD is crucial to optimize treatment plans and enhance treatment safety.

**Objective:**

This study aims to evaluate the medication preferences among patients with multiple chronic diseases in rural areas of an eastern province of China.

**Methods:**

A discrete choice experiment (DCE) was used to measure patients’ medication preferences. According to literature research, expert panel discussions, and in-depth patient interviews, we identified six attributes: monthly out-of-pocket cost, onset speed of action, adverse effects, whether it is covered by health insurance, origin of medications, and types of medications. The conditional logit models (CLM) and mixed logit models (MIXL) were used to evaluate the choice data. Willingness to pay (WTP) was used to reflect the monetary value that patients were willing to pay or receive reimbursement after changes in different attribute levels.

**Results:**

A total of 956 respondents were included in the analysis. Of which, 68.62% were female, with an average age of 68 years, and 65.89% had a Body Mass Index (BMI) greater than or equal to 24. Statistical significance was observed for all attributes (*p* < 0.001). The preferred medication for patients encompassed low monthly out-of-pocket costs, rapid onset of action, rare adverse effects, and a preference for Western medicine, health insurance-covered medication and domestic medication. The onset speed of action was a primary consideration for patients, who demonstrated a willingness to pay an additional CNY151.37 per month for a medication with a rapid onset of action.

**Conclusion:**

Rural patients with multiple chronic diseases preferred medications with rapid onset, rare adverse, Western medications, domestic medication, and health insurance-covered medication. Medical staff can effectively combine the Health Belief Model (HBM) to help patients with multiple chronic diseases improve their confidence and understanding of medication selection, to improve their health management.

## 1 Introduction

In 1970, Feinstein (1) first proposed the concept of “Comorbidity,” and in 2008, WHO defined this phenomenon as “multi-chronic disease” (Multimorbidity) (2), that is, patients suffer from two or more chronic diseases at the same time (3). Multiple chronic diseases are anticipated to rise in global prevalence (4). Studies indicate that approximately one in three individuals worldwide is affected by an multiple chronic diseases, with this figure experiencing a significant upward trend (5, 6). According to a recent national survey, it is estimated that around 45% of China’s elderly population, aged 65 and above, are afflicted with multiple chronic diseases (7). Furthermore, China is undergoing a swift demographic aging process. The proportion of the elderly population (60 years and older) will increase from 12.4% in 2010 to 28% in 2040 (8). It is foreseeable that the prevalence of multiple chronic diseases will increase significantly in the future, placing a huge burden on the utilization and cost of healthcare services in China (9). Compared to a single chronic disease, patients with multiple chronic diseases face a higher risk of death (10) and need to pay more for healthcare (11, 12), and quality of life is also affected (13). Furthermore, patients with multiple chronic diseases confront an additional challenge: polypharmacy, which can escalate the risk of adverse drug events, side effects, and drug–drug interactions, among a multitude of other potential complications (14).

Understanding the specific drug preferences of patients with multiple chronic diseases is critical to optimizing treatment regimens and improving treatment safety. Each patient assigns different importance to various aspects of medication therapy. For instance, some patients may emphasize the benefits of medication, while others may be more concerned about the risks of medication (15). Therefore, it is important for healthcare professionals, including physicians and pharmacists, to thoroughly understand and consider the individual preferences of patients with multiple chronic diseases to tailor treatment plans effectively. Incorporating patient preferences into clinical care can help us get information that cannot be obtained through clinical trial data, and can be informative for improving patient medication adherence and patient satisfaction.

Currently, more and more national health administrations are paying attention to patients’ medication selection preferences in healthcare decision-making (16–18). In recent years, The Centre for Drug Evaluation of the State Drug Administration of China (CDE) has also issued several guidelines, emphasizing patient-centeredness and the importance of taking patient preferences into account in prescribing decision-making (19, 20).

Discrete Choice Experiment (DCE) is based on the attribute value theory and random utility theory proposed by Lancaster (21), which simulates a rational, direct, and near-realistic decision-making process. It can simultaneously consider the comprehensive impact of multiple factors on the choice outcome, and can more accurately elicit individual preferences compared to other preference research methods (22). DCE has been applied in the study of medication preferences of patients with chronic diseases at home and abroad. For example, van Heuckelum et al. (23) investigated the preferences for anti-rheumatic drugs among patients with rheumatoid arthritis in the Netherlands. Holmes (24) conducted a study on medication preferences among patients with hypertension across nine European countries. Liu et al. (25) explored the preferences of anti-hyperglycemic medications among patients with type 2 diabetes mellitus. Lv et al. (26) studied the medication preferences of patients with type 2 diabetes mellitus. In summary, most previous studies about medication preference for chronic diseases mainly focus on the medication for a certain disease and have not yet been conducted on patients with multiple chronic diseases. Studies have found that the prevalence of chronic diseases and comorbidities among middle-aged and elderly people in rural areas of China is relatively serious (27), especially in rural areas with underdeveloped medical conditions, the compliance of comorbidities is poor (28). Based on the differences between rural and urban areas in culture, economy, and medical and health services, patients with chronic diseases may also have different preferences for drug selection (29). Therefore, this study investigated the medication choice preferences of patients with multiple chronic diseases in rural areas of an eastern province in China, to fill the gap in this research area and provide references and lessons for pharmacy services for patients with multiple chronic diseases in rural areas.

## 2 Methods

### 2.1 Discrete choice experiment

DCE is based on the random utility theory, where respondents are asked to choose the option that offers them the greatest utility in a given situation (30). In DCE, researchers often design choice sets with various combinations of attributes and levels, asking respondents to make trade-offs between these options (31). This study was designed and analyzed concerning the Guidelines for the Application of DCE in Health Services (32) and the content published by the International Society for Pharmacoeconomics and Outcomes Research (ISPOR) (33).

### 2.2 DCE design

A combination of qualitative and quantitative methods was used to derive attributes and levels according to the study guidelines (31). By reviewing the literature, we identified attributes related to patients’ medication preferences in existing DCE, Best-Worst Scaling (BWS), and other related studies. We developed an initial list of seven attributes, including monthly out-of-pocket cost (34–36), onset speed of action (25, 37, 38), adverse effects (39–42), daily pill count (43, 44), daily dosing frequency (45), duration of action (37) and Whether it is covered by health insurance (46–49). We then convened a group of experts for interviews, during which they suggested additional attributes and eliminated those considered less valuable. After discussion, we retained four key attributes: monthly out-of-pocket cost, onset speed of action, adverse effects, and whether it is covered by health insurance. We also proposed adding a distinction between Chinese medicine and Western medicine as an attribute. Concurrently, we conducted in-depth interviews with patients. Based on the interview data, we found that the origin of medications, whether domestic or imported, significantly impacted the medication selection of patients with multiple chronic diseases. Therefore, we added the attribute of domestic or imported medications to the list.

The monthly out-of-pocket cost was set at four levels, with the lowest cost representing the cost of medications for hypertensive diseases and the highest cost representing the cost of medications for cardiovascular diseases. The onset speed of action was set at two levels: Rapid onset and Stable onset. Rapid onset indicates that after treatment with the medication, patients can feel a significant improvement in symptoms within a few hours to 2 weeks. Stable onset means that after the use of medication treatment, usually within weeks to months can alleviate some chronic symptoms. Adverse effects were set at two levels. Rare adverse effects mean that adverse effects caused by the medication are very rare, and patients will hardly experience any significant adverse reactions. Minimal adverse effects mean that adverse effects occur infrequently and usually do not have a serious effect on the patient. A list of these attributes and levels is presented in Table 1.

**Table 1:** Attributes and levels in the DCE.

Attributes	Levels
Monthly out-of-pocket cost	CYN 10
	CYN 50
	CYN 100
	CYN150
Onset speed of action	Stable onset
	Rapid onset
Adverse effects	Minimal adverse
	Rare adverse
Types of medications	Traditional Chinese medication
	Western medication
Origin of medications	Domestic medication
	Imported medication
Whether it is covered by health insurance	Health insurance-covered medication
	Non-Health insurance-covered medication

Determining attributes and levels is a crucial step in DCE research. In this study, including six attributes and their corresponding levels (2^5^*4^1^) will result in 128 scenarios combined into 8,128 choices. D-efficient design with SAS software (version 9.4), generating 16 choice sets. To reduce the cognitive load on patients, the 16 choice sets were split into 2 versions. Each respondent was randomly assigned one of the 2 blocks that required 9 DCE questions. A duplicate choice set (Scenario 3 = Scenario 9) was added to each version of the questionnaire to test for internal consistency within the questionnaire. That is, when there was inconsistency between the patient’s choices for scenario 3 and scenario 9, the patient was considered not to have understood the questionnaire and should be excluded from the analysis. In a series of medication choice questions, respondents were asked to choose between two hypothetical medications. Each choice set consists of two alternatives (medication A and medication B) and asks the question: Which of the above two medications do you prefer? An example of a choice task is shown below in Table 2.

**Table 2:** An example of DCE choice set.

Attributes	Medication A	Medication B
Monthly out-of-pocket cost	CNY10	CNY50
Onset speed of action	Rapid onset	Stable onset
Adverse effects	Minimal adverse	Rare adverse
Types of medications	Traditional Chinese medication	Western medication
Origin of medications	Imported medication	Domestic medication
Whether it is covered by health insurance	Health insurance-covered medication	Non-health insurance-covered medication
Which of the above two medications do you prefer?	**□**	**□**

### 2.3 Development of questionnaire

To evaluate the questionnaire’s efficacy, the research group initially conducted a preliminary survey with 30 patients who have multiple chronic diseases. Subsequently, aiming to enhance the survey’s clarity and functionality, the questionnaire was refined based on the feedback and issues identified during the preliminary survey. This iterative process ensured that the questions were acceptable to respondents and that the survey instrument was both reasonable and operable. Ultimately, the questionnaire was divided into 2 versions, each including three parts. The first part is the sociodemographic survey information of patients with multiple chronic diseases, including gender, age, education level, family income, and type of health insurance. The second part is a survey of the prevalence of multiple chronic diseases, including the number of chronic diseases suffered, disease duration, number of medications taken, and self-assessed health status. The third part is the DCE choice set, with nine choice sets for each version of the questionnaire.

### 2.4 Data collection

This study employed a multi-stage stratified random sampling method to conduct the survey, with data collection taking place over a period from September to December 2023. First of all, according to the high, medium, and low levels of per capita GDP, we selected three prefecture-level cities in an eastern province of China. Then in each prefecture-level city, we selected three counties according to the same principle. In each county, we selected four townships (streets) based on the same principle. Six villages were randomly selected in each township. In each village, 10–15 residents were randomly selected for the survey. The respondents were recruited by family doctors and gathered in the meeting room of the village clinic for investigation. Respondents were informed in advance that participation in the survey was anonymous and voluntary, and their informed consent was obtained prior to the survey. It took about 15 min for each respondent to complete the questionnaire, and each respondent participated in the survey independently. A total of 2,432 questionnaires were sent out and 2,415 valid questionnaires were recovered, with an effective recovery rate of 99.3%. The minimal sample size was required using a rule-of-thumb formula for DCE studies suggested by Orme (50). Therefore, the minimum sample size calculated in this study is not less than 125.

### 2.5 Criteria for inclusion and exclusion

Inclusion criteria were: (1) rural patients who were diagnosed by a doctor as suffering from two chronic diseases or more; (2) suffering from at least two diseases (the diseases included in this study were hypertension, diabetes mellitus, dyslipidemia, arthritis, rheumatism, cardiovascular diseases, cerebral vascular diseases, chronic lung diseases, digestive diseases, urinary diseases, and psychiatric disorders); and (3) those who were able to clearly express their thoughts and situations and had good communication skills.

Exclusion criteria were: (1) patients with non-chronic co-morbidities or unclear diagnoses; (2) those with specific medication inaccuracies; (3) those with mental disorders or cognitive disorders, those with hearing loss; and (4) those with severe or terminal illnesses. Ultimately, a total of 1,070 study samples were included in this study.

Ethical approval for the patient preference study was granted by the Medical Ethics Committee, Weifang Medical University, Shandong Province, China, approval number 2021YX-066.

### 2.6 Statistical analysis

SPSS 27.0 and Stata 17.0 were used for statistical analyses. Descriptive statistics were used to present the sociological characteristics of the respondents. Conditional logit model (CLM) and mixed logit model (MIXL) were used to analyze the data of DCE. The regression model suitable for this study was determined by Akaike Information Criterion (AIC) and Bayesian Information Criterion (BIC). The magnitude and direction of the model regression coefficients reflect the magnitude and direction of the influence of each attribute on respondents’ service utilization preferences. Positive regression coefficients signify a favorable service preference among respondents for the respective attribute, indicating a preference; conversely, negative regression coefficients denote an unfavorable service preference, indicating an aversion.

Relative Importance (RI) is a measure of the magnitude of the difference that each attribute produces in the total attribute preference, this difference is the range of preference weights for the attribute, and the attribute importance is calculated in terms of the range of horizontal relative weights to obtain a set of attribute relative importance values that sum up to 100%. The higher the score, the more important the attribute is to the respondent.

Initially, all attributes were encoded as dummy-coded categorical variables. However, in the MXL and the Willingness to Pay (WTP) calculations, the cost attribute was treated as a continuous variable. WTP is used to reflect the monetary value that patients are willing to pay or reimburse for changes in the level of different attributes. A positive sign indicates the additional monthly out-of-pocket cost that respondents are willing to incur to receive that level of medication, and a negative sign indicates the additional reimbursement that would be required for respondents to receive that level of medication.

## 3 Results

### 3.1 Participants

Of the 1,070 participants included in the study, 114 participants did not pass the internal consistency test. To ensure the accuracy of the results, we excluded them and finally analyzed the remaining 956 respondents. Of the 956 respondents, 68.62% of them are female, the median age is 69 years, and 65.89% of them have a Body Mass Index (BMI) greater than or equal to 24. This is consistent with the estimated prevalence of patients with multiple chronic diseases in China, indicating that the sample has good national representativeness (51). 82.64% of them were married, 74.90% of them were unemployed or underemployed, about 61.14% of them were in primary school and below, 45.29% of them had annual family income less than CNY10000, and 95.82% of them were urban resident basic medical insurance. In the study population, 57.74% of them had two chronic diseases, 73.22% of the participants’ diseases lasted for 5 years or more, and 33.89% of them were using two medications. Prevalent chronic diseases included hypertension (34.94%), cardiovascular diseases (16.43%), diabetes mellitus (18.14%), and dyslipidemia (9.44%). Other conditions are detailed in Table 3. The types of multiple chronic diseases are detailed in Appendices 1, 2.

**Table 3:** Characteristics of study participants (*N* = 956).

Characteristics	All	%
Age/years, mean(SD)[range]: 68.03(±8.85)[28–90] Median: 69		
Sex		
Female	300	31.38
Male	656	68.62
Marital status		
Unmarried	6	0.62
Married	790	82.64
Widowed or divorced	160	16.74
Employment status		
Employed	184	19.25
Retired	56	5.85
Unemployed	716	74.90
Education level		
Primary school and below	661	61.14
Junior high school and above	295	30.86
Annual family income/CNY		
≤10,000	433	45.29
10,001–25,000	138	14.44
>25,000	385	40.27
BMI, mean (SD)[range]:25.56(±3.57)[16.16–36.98]		
Medical insurance type		
Urban employee basic medical insurance	22	2.30
Urban resident basic medical insurance	916	95.82
Others	18	1.88
Number of chronic diseases		
2	552	57.74
3	281	29.39
≥4	123	12.87
Time since diagnosis/years		
≤1	36	3.77
1–5	220	23.01
5–10	349	36.51
>10	351	36.71
Number of medications currently taking for hypertension		
≤1	151	15.80
2	324	33.89
3	235	24.58
≥4	246	25.73
Types of chronic diseases		
Hypertension	840	34.94
Diabetes mellitus	436	18.14
Dyslipidemia	227	9.44
Arthritis or rheumatism	222	9.24
Cardio vascular diseases	395	16.43
Cerebral vascular diseases	112	4.66
Chronic lung diseases	51	2.12
Digestive diseases	84	3.49
Urinary diseases	13	0.54
Psychiatric disorders	4	0.17
Other chronic diseases	20	0.83

### 3.2 Patients’ preferences

The results showed that the AIC and BIC values of the MIXL were significantly better than those of the CLM, and there were no substantial differences in the preference results between the two models in Appendix 3. The regression results of the MIXL for medication preference of patients showed that the six attributes of monthly out-of-pocket cost, onset speed of action, adverse effects, origin of medications, whether it is covered by health insurance, and types of medications had a significant effect on the medication choice preference of patients.

Specifically, patients exhibit a preference for medications that are rapid onset, with rare adverse, western medications, domestic medications, and health insurance-covered medications when making their choices. Among the non-economic factors, “rapid onset” has the greatest utility in patients’ medication selection (*β* = 2.091), followed by “western medication” (*β* = 0.670) and “health insurance-covered medication” (*β* = 0.523). The least influential factors are “Rare adverse” (*β* = 0.382) and “domestic medication” (*β* = 0.244). The monthly out-of-pocket cost for medication also impacts the choice of medication (*β* = −0.014), albeit negatively (see Table 4).

**Table 4:** Mixed logit model results (*N* = 956).

Attributes/Level	β	SE	P	SD	SE	P
Onset speed of action (ref: Stable onset)						
Rapid onset	2.091	0.174	<0.001	3.598	0.223	<0.001
Adverse effects (ref: Minimal adverse)						
Rare adverse	0.382	0.064	<0.001	−0.492	0.132	<0.001
Types of medications (ref: Traditional Chinese medication)						
Western medication	0.670	0.117	<0.001	2.820	0.170	<0.001
Origin of medications (ref: imported medication)						
Domestic medication	0.244	0.059	<0.001	−0.559	0.116	<0.001
Whether it is covered by health insurance (ref: Non-Health insurance-covered medication)						
Health insurance-covered medication	0.523	0.108	<0.001	2.403	0.171	<0.001
Monthly out-of-pocket cost	−0.014	<0.001	<0.001			
Constant	0.005	0.061	0.928			
n	956					
Observation 17,120(956*16)						
Log-likelihood −3433.950						
AIC 6891.900						
BIC 6983.496						

SE, Standard Error; SD, Standard Deviation; AIC, Akaike Information Criterion; BIC, Bayesian Information Criterion.

### 3.3 Attribute relative importance

**Figure 1:**
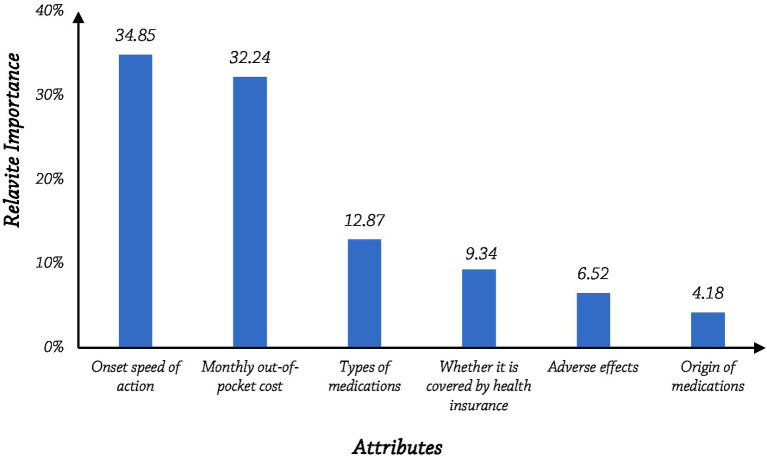
The relative importance score of each attribute.

The relative importance of an attribute is determined by the proportion it represents when its range is divided by the sum of ranges for all attributes. The onset speed of action had the greatest relative importance (34.85%), followed by monthly out-of-pocket cost (32.24%), and these two attributes were the most important determinants of medication preference in patients. This was followed by types of medications (12.86%) and whether it is covered by health insurance (9.34%). The relative importance of adverse effects (6.52%) and origin of medications (4.18%) was minimal (see Figure 1).

### 3.4 Willingness to pay

The regression results of patients’ preferences for medications, estimated willingness to pay, and 95% confidence intervals are shown in Table 5. The three attributes with higher willingness to pay for patients are onset speed of action, types of medications, and whether it is covered by health insurance, and the last two are adverse effects and origin of medications. Patients were willing to pay CNY151.37 (US$21.35) per month for rapid onset medications and were willing to pay CNY55.87 (US$7.88) per month for western medications. In addition, in exchange for health insurance-covered medications, patients were willing to pay CNY40.58 (US$5.72) per month, and in exchange for rare adverse medications, patients were willing to pay CNY28.30(US$3.99) per month. Compared with imported medications, patients were willing to pay CNY18.15 (US$2.56) per month.

**Table 5:** Willingness to pay of each attribute and level.

Attributes/Levels	WTP (CNY)	95%CI	*P*
Onset speed of action (ref: Stable onset)			
Rapid onset	151.37	(125.86 ~ 176.85)	<0.001
Adverse effects (ref: Minimal adverse)			
Rare adverse	28.30	(20.29 ~ 36.31)	<0.001
Types of medications (ref: Traditional Chinese medication)			
Western medication	55.87	(38.99 ~ 72.75)	<0.001
Origin of medications (ref: Imported medication)			
Domestic medication	18.15	(10.08 ~ 26.23)	<0.001
Whether it is covered by health insurance (ref: Non-Health insurance-covered medication)			
Health insurance-covered medication	40.58	(25.83 ~ 55.32)	<0.001

WTP space were in RMB. 1US$ = 7.09 RMB; CI, Confidence Interval.

## 4 Discussion

According to the results of the study, rural patients with multiple chronic diseases preferred medications with rapid onset, rare adverse, Western medications, domestic medication, and health insurance-covered medication. The Health Belief Model (HBM) provides a powerful theoretical framework to explore the medication use behavior of patients with multiple chronic diseases. The model suggests that individuals’ adoption of health behaviors is related to their perceived susceptibility, perceived severity, perceived benefits, perceived barriers, cues to action, and self-efficacy (51). Therefore, we use the theory of HBM to explore the medication behavior of patients with multiple chronic diseases.

Rapid onset of action has the most significant impact on medication preferences among rural patients with multiple chronic diseases. The study found that the onset speed of action was ranked first among all attributes in the choice of medication by patients. This is similar to several DCE studies in China (38, 52). The misconception that medication should completely cure the disease in a short period is a misunderstanding in the minds of many patients with chronic diseases, who hope that the medication will cure the disease and have high expectations of the medication (53). This expectation reflects patients’ awareness of their own health status and inner anxiety, demonstrating their awareness of chronic disease susceptibility. This awareness influences their understanding of the disease, aligning with the construct of perceived susceptibility in HBM. Since patients need to take medication for a long period or even for a lifetime, without stable financial income and medical insurance, it will impose a heavy financial burden on themselves and their families, so patients will be more likely to stop taking medication halfway (54). Therefore, many patients have a “symptom-driven treatment,” which means that they use medication when their symptoms worsen and stop the medication when their symptoms decrease to end the process of disease treatment by obtaining medication with rapid onset. This is an important reason why patients tend to favor medications that are rapid onset. Patients may realize that they are at risk of multiple chronic diseases, so they tend to choose medications that can work quickly to relieve the pain as soon as possible. This was linked to their perception of threats to their health, indicating a desire to reduce health risks through effective treatment, demonstrating the construct of perceived severity in HBM.

Health insurance-covered medications have a greater impact on medication choice preferences among rural patients with multiple chronic diseases. Whether it is covered by health insurance has a greater impact on the medication choices of patients. Patients may give up Non-health insurance-covered medication in the process of medication selection in favor of less burdensome health insurance-covered medication. This result is consistent with previous studies (46, 48). At the end of 2023, the National Health Insurance Bureau and other departments introduced *the National Drug Catalogue for Basic Medical Insurance, Workers’ Compensation Insurance, and Maternity Insurance (2023)*, with 126 new types of medications, bringing the total number to 3,088, which, overlaid with price reductions and reimbursement by health insurance, will reduce the burden on patients by more than 200 billion yuan (55). In recent years, the proportion of medications used by health insurance in medical institutions has increased year by year, and the price level of commonly used medications has dropped significantly, which, together with the increasing reimbursement rate of health insurance, has reduced the burden of medications on patients. In addition, the medication has been approved by the medical insurance department to enter the medical insurance catalog, and its clinical effectiveness and safety meet the requirements. Compared with non-health insurance-covered medication, it is more reliable, so most patients prefer to choose health insurance-covered medications. Rural patients with multiple chronic diseases tend to choose Health insurance-covered medications, reflecting the construct of perceived benefits in HBM. At the same time, Health insurance-covered medications can reduce their financial burden, which increases their understanding of the potential benefits of using these medications.

Western medications are an important factor in the medication choice preferences of patients with multiple chronic diseases. This study found that patients were more inclined to choose Western medication when choosing medications. And to obtain Western medications, patients were willing to pay CNY55.87 for them. Relevant non-DEC studies in China have shown that most patients with chronic disease will choose western medications (56). At the primary level, the availability of Western medication is better than that of traditional Chinese medication. Firstly, family doctors and pharmacists in primary care are usually more familiar with the use and formulation of Western medication, so the use of Western medications is more common. This reflects the construct of cue to action in HBM and serves as a guide for healthcare professionals in recommending medication to patients, leading to a preference for Western medication. Secondly, traditional Chinese medications are relatively decentralized in terms of production and supply, and there are complexities in the procurement and processing of different herbs. This leads to a result that the availability of traditional Chinese medication is less accessible than that of western medications. Studies have shown that there is a large difference in the number of proprietary traditional Chinese medications and Western medications in second - and third-level medical institutions. The number of Western medications in tertiary medical institutions is 2.25 times that of proprietary traditional Chinese medication. The number of Western medications in secondary medical institutions is 2.43 times that of proprietary Traditional Chinese medication. Compared with Traditional Chinese medication, Western medications are easier to obtain (57). In addition, compared with Western medications, traditional Chinese medications are more complicated to take, and some of them need to be decocted. Which is not only a hassle for patients to take daily but also imposes stringent requirements for the time of decocting and the amount of medicine to be used. It is difficult for patients to fully comply with the doctor’s instructions, and increased their burden of medication. Compared with traditional Chinese medication, the availability of Western medication is higher, and the method of administration is simpler, eliminating patients’ obstacles related to the complexity and difficulty of taking medication. This reflects the construct of perceived barriers in HBM.

Adverse effects and imported versus domestic medication have a low impact on the medication choice preferences of rural patients with multiple chronic diseases. Adverse effects are attributes that have a low degree of influence on medication preference. Overseas DCE studies have shown that the risk of medication side effects is not a concern for some chronic diseases in drug therapy (39, 44, 58). The reason for this may be related to the lack of knowledge about safe medication use among patients. According to the present study, the respondents’ education level was low, and due to their cultural cognitive level, many patients lacked knowledge about safe drug use and paid little attention to the interactions among drugs and the adverse drug reactions caused by drugs. To choose medications with rare adverse, rural patients with multiple chronic diseases are willing to pay CNY28.30 for this. Choosing a medication with rare adverse reflects the patient’s concern about potential side effects, which is a direct response to the construct of perception barriers in the HBM. To address these barriers, patients are more likely to choose medications that are clinically considered safe.

We found that rural patients with multiple chronic diseases preferred domestic medications and were willing to pay CNY18.15 additional compared with imported medications. A non-DCE study found that there was no statistical difference in adverse effects between domestic and imported medications (59, 60). Over a treatment cycle, patients had a higher preference for domestic medications relative to imported medications because of less perceived expense, higher trust, and the perception that the medications were more in line with the national constitution. In a treatment cycle, compared with imported medications, patients perceive that domestic medications cost less, trust more, and think that medications are more in line with the constitution of Chinese people. This behavior reflects their understanding and confidence in managing their health and embodies the construct of self-efficacy in the HBM.

According to the theory of HBM, we propose a variety of measures to improve the medication behavior of patients with multiple chronic diseases. Firstly, we should improve the pharmaceutical care ability of healthcare professionals, strengthen the pharmaceutical guidance for patients with multiple chronic diseases, help patients establish scientific medication awareness, and improve their medication behavior. This can make the patient aware of the risks to their health and the potential seriousness of the disease. Secondly, it enhances communication between patients and medical professionals and promotes decision-making sharing. In the process of pharmaceutical service, medical staff should customize personalized pharmaceutical service programs for patients according to their medication experience and preference information. This can enhance patients’ confidence in medication and improve patients’ medication compliance. In addition, medical staff should identify and alleviate patients’ medication barriers, so that patients can participate more actively in treatment decision-making. Furthermore, we should promote the reform of the medical insurance system, improve the level of welfare, reduce the economic burden of patients’ medication, and reduce the withdrawal and deviation of medication caused by the economic burden. To further enhance patients’ awareness of the benefits of medication and improve their medication compliance.

## 5 Limitations

This study fills a significant gap in our understanding of medication choice preferences in patients with multiple chronic conditions. Nevertheless, certain limitations remain. First of all, the included samples were only from a certain province in China. Due to the obvious regional characteristics of the prevalence of multiple chronic diseases, the influence of regional factors should be taken into account when extrapolating the research conclusions. Future studies should be carried out according to regions to obtain representative samples across the country. Second, due to the inherent limitations of the DCE, only six attributes, namely, out-of-pocket drug costs, onset speed of action, adverse effects, types of medications, origin of medications, and whether it is covered by health insurance, were included in analyzing the preference for multiple chronic diseases, and the influence of other attributes on the preference for multiple chronic diseases could not be reflected. Finally, although this study took effective measures throughout the experimental design and survey to present respondents with real choice scenarios to the greatest extent possible to ensure the accuracy of preference information collection, the DCE investigated patients’ stated preferences, and further validation whether and to what extent their actual medication choice behaviors are consistent is still needed for more studies in the future.

## 6 Conclusion

In conclusion, patients with multiple chronic diseases in rural areas preferred rapid onset, health insurance-covered medication, and Western medications when making their medication selections. These findings fill the evidence of medication selection preferences of rural patients with multiple chronic diseases, which can guide family physicians in providing treatment plans and improve medication satisfaction and experience of patients with multiple chronic diseases, and the findings are of strong guiding significance.

## Data Availability

The raw data supporting the conclusions of this article will be made available by the authors, without undue reservation.

## Appendix

Appendix 1 The main combination of two chronic diseases (*N* = 552).

**Table:**

Multiple chronic disease combinations	N	%
Hypertension+diabetes mellitus	163	29.53
Hypertension+cardio vascular diseases	133	24.09
Hypertension+arthritis, rheumatism	62	11.23
Hypertension+dyslipidemia	48	8.70
Hypertension+cerebral vascular diseases	32	5.80

Appendix 2 The main combination of three chronic diseases (*N* = 281).

**Table:**

Multiple chronic disease combinations	N	%
Hypertension+diabetes mellitus+cardio vascular diseases	51	18.15
Hypertension+diabetes mellitus+dyslipidemia	43	15.30
Hypertension+diabetes mellitus+arthritis, rheumatism	29	10.32
Hypertension+dyslipidemia+arthritis, rheumatism	23	8.19
Hypertension+arthritis, rheumatism+cerebral vascular diseases	16	5.69

Appendix 3 Conditional logit model (*N* = 956).

**Table:**

Attributes/Levels	β	SE	*P*
Onset speed of action (ref: Stable onset)			
Rapid onset	0.618	0.025	<0.001
Adverse effects (ref: Minimal adverse)			
Rare adverse	0.172	0.025	<0.001
Types of medications (ref: Traditional Chinese medication)			
Western medication	0.267	0.026	<0.001
Origin of medications (ref: Imported medication)			
Domestic medication	0.073	0.026	0.005
Whether it is covered by health insurance (ref: Non-Health insurance-covered medication)			
Insurance-covered medication	0.153	0.025	<0.001
Monthly out-of-pocket cost	−0.005	<0.001	<0.001
Constant	−0.005	0.027	0.850
n	956		
Observation	17,120(956*16)		
Log-likelihood	−4696.048		
Psedo R2	0.112		
AIC	9406.095		
BIC	9459.526		
